# The regulatory mechanism and biological significance of the Snail-miR590-VEGFR-NRP1 axis in the angiogenesis, growth and metastasis of gastric cancer

**DOI:** 10.1038/s41419-020-2428-x

**Published:** 2020-04-17

**Authors:** Bin Mei, Jiajie Chen, Ni Yang, Yang Peng

**Affiliations:** 1 Hepatic Surgery Centre, Tongji Hospital, Tongji Medical College, Huazhong University of Science and Technology, 430030 Wuhan, China; 2 Department of Geriatrics, Tongji Hospital, Tongji Medical College, Huazhong University of Science and Technology, 430030 Wuhan, China

**Keywords:** Cancer, Non-coding RNAs

## Abstract

Vascular endothelial growth factor receptor (VEGFR) and neuropilins (NRPs), a co-receptor of VEGF, play a key role in the formation and development of blood vessels and in tumour growth and metastasis. However, whether VEGFR1/2 and NRP1 are regulated by the same upstream mechanism is unclear, especially in gastric cancer. We used prediction tools to detect miRNAs that may simultaneously regulate VEGFR1/2 and NRP1, and we finally determined that miR-590 can simultaneously regulate VEGFR1/2 and NRP1 in gastric cancer. We discovered that miR-590 was downregulated in gastric cancer tissues and cell lines, and this was related to the dysregulation of the transcription factor SNAIL. In addition, the overexpression of miR-590 inhibits the migration, invasion, proliferation and D-MVA levels of gastric cancer cells in vivo and in vitro by targeting VEGFR1/2 and NRP1. We also demonstrated that miR-590 may be a useful marker for the prognosis of gastric cancer with Kaplan–Meier survival analysis. Since the epithelial-to-mesenchymal transition (EMT) is an important mechanism of tumour invasion and metastasis and VEGFR1/2 and NRP1 can promote the occurrence of EMT, we speculated that miR-590 can regulate the occurrence of EMT. Immunoblot and immunofluorescence analyses confirmed that the overexpression of miR-590 can inhibit the EMT in gastric cancer cells. Since SNAIL is also a mesenchymal marker, our results revealed a new, positive feedback loop. As a transcription factor, SNAIL inhibits the expression of miR-590, thereby upregulating the expression levels of NRP1 and VEGFR1/2; this leads to the development of EMT in gastric cancer and the upregulation of SNAIL.

## Introduction

Gastric cancer (GC), a major worldwide health problem, has remained the fifth most common cancer type and the third most common cause of cancer-related mortality since 2012^1^. The leading cause of death in GC is distant metastasis, which relies on tumour angiogenesis^2^. The occurrence of any solid tumour is based on angiogenesis. Tumour angiogenesis is the result of multiple angiogenesis factors. Among these angiogenesis factors, vascular endothelial growth factor (VEGF) and its receptor, VEGFR, are essential for tumour angiogenesis^3,4^.

VEGFR contains three subtypes, namely, VEGFR-1 (also known as FLT-1), VEGFR-2 (also known as KDR) and VEGFR-3; VEGFR-1 and VEGFR-2 are primarily expressed in vascular endothelial cells, and VEGFR-3 is highly expressed in lymphatic endothelial cells^5^. Recent studies have shown that VEGFR-1 and VEGFR-2 are expressed not only in endothelial cells but also in many tumour cells^6,7^. In addition, VEGFR1/2 has been shown to be associated with tumour distant metastasis^8–10^. These results fully show the correlation between VEGFR1/2 and tumour development and progression.

As a co-receptor of VEGFR, neuropilins (NRPs) play a key role in the formation and development of blood vessels and in tumour growth and metastasis^11^. NRPs contain the following two subtypes: NRP1 is mainly expressed in vascular endothelial tissue, and NRP2 is mainly expressed in lymphatic epithelium^12,13^. Further studies have found that NRP1 can still promote tumour angiogenesis, accelerate the growth and proliferation of tumour cells, and inhibit the apoptosis of tumour cells in the absence of VEGFR^14^; this may indicate that NRP1 can form co-receptors with some non-VEGFR growth factor receptors^15,16^.

The upstream regulatory mechanisms of VEGFR 1/2 and NRP1 are diverse. Higgins KJ et al. found that SP1, SP3 and SP4 regulate the expression of VEGFR2 by binding to the GC-enriched region of the VEGFR2 promoter^17^. Kim J et al. identified that the abnormal expression of VEGFR 1/2 is related to abnormal promoter methylation levels^18^. QuenTmeier H et al. identified that DNA methylation will silence the expression of VEGFR 2/3 in umbilical vein endothelial cells^19^. In terms of the regulatory mechanism of NRP1, the current research focuses on the transcription factors sp1/3 and AP1. Studies have shown that the acetylation of sp1/sp3 histone significantly inhibited the expression of NRP1, and NRP1 is the downstream target of the transcription factors AP1 and Ets-1^20^. These results showed that the expression of NRP1 and VEGFR 1/2 is regulated by genes and epigenetic inheritance, among other factors. NRP1, a co-receptor of VEGFR 1/2, may be regulated by some upstream regulatory mechanism together with VEGFR 1/2, but there is currently no relevant research on this mechanism.

MicroRNAs (miRNAs) are small noncoding RNAs, 19–25 nt long, that typically regulate downstream gene expression and play important roles in cell-cycle regulation, differentiation, apoptosis and tumour development^21^. MiRNAs have the function of network regulation; that is, the same miRNA can regulate the expression of multiple genes. Therefore, we speculate that there may be a certain miRNA that can regulate the expression of NRP1 and VEGFR 1/2 at the same time. In this study, we found this miRNA, namely, miR-590. Then, we examined the expression of miR-590 and the prognostic potential of miR-590 in GC. To investigate the dysregulation of miR-590 in GC, we investigated its upstream genes. Finally, we studied the effect of miR-590 on the angiogenesis, growth, proliferation, invasion and metastasis of GC by targeting VEGFR1/2 and NRP1.

## Materials and methods

### Clinical data and cell lines

This study collected 118 fresh human tissues, including GC specimens and adjacent normal mucosal tissues (at least 1 × 1 cm^2^ tissues per sample), that underwent surgery in Wuhan Tongji hospital from 2014 to 2015. All patients must meet the inclusion criteria: (1) Patients aged >20 years; (2) Diagnosis of GC was inspected by gastric endoscopy. Meanwhile, gastric nonepithelial malignant tumours or benign gastric tumours such as gastric stromal tumour patients will be excluded. The study was in accordance with the Ministry of Health ‘Biomedical Research Involving Human Ethics Review (Tentative)’ and the Declaration of Helsinki on Ethical Principles for Medical Research Involving Human Subjects. All GC patients provided written informed consent to collect and use samples, and the medical ethics committee of Tongji Hospital at Tongji Medical College of the Huazhong University of Science and Technology approved the experiments.

GES-1 (a non-tumour gastric cell line) was purchased from the Type Culture Collection of the Chinese Academy of Sciences (Shanghai, China), MKN-45, AGS (cell lines of GC) were obtained from the American Type Culture Collection (ATCC; Manassas, VA, USA). Those three cell lines has been authenticated by STR profiling and tested for mycoplasma contamination. All cell lines were incubated in a humidification incubator at 37 °C with 5% CO2 according to ATCC protocols. Cells were grown in RPMI1640 (HyClone, Logan, Utah, USA) containing 10% foetal bovine serum (FBS).

### RNA extraction and miRNA detection

Total miRNAs in cultured cells and human tissue samples were extracted with RNAiso for Small RNA (TaKaRa Bio, Otsu, Japan) following the manufacturer’s procedures. The addition of poly-a tails to miRNA and U6 was accomplished by the miRNA reaction buffer mixture (TaKaRa Bio). The total miRNA of 5 ng was then reversely transcribed into cDNA using the miRNA PrimeScript RT enzyme Mix (TaKaRa Bio). Real-time PCR was performed with SYBR® Premix Ex Taq™ II (TaKaRa Bio) in a CFX96™ Real-Time PCR Detection System (Bio-Rad). The PCR procedure was 95 °C for 30 s, followed by 40 cycles of 95 °C for 5 s and 60 °C for 30 s. Normalise data based on U6 snRNA data. The melting curve was analysed to determine the specificity of the products after amplification. Cycle threshold (Ct) values were calculated using SDS 2.0 software (Applied Biosystems) to evaluate miRNAs’ expression levels. Relative to the U6 small nuclear RNA (RNU6B), the 2-ΔΔCt method was used to normalise the concentration of tissue or cell line samples. The Ct values of target miRNAs subtract the corresponding Ct values of RNU6B is worth to the value of ΔCt. Then with the ΔCt value of the cancer samples minus the ΔCt of the control samples got the value of ΔΔCt. Using the equation 2-ΔΔCt to calculate the change of gene expression.

### Oligonucleotide transfection

miRNA mimics and negative control were synthesised by Sangon Biotechnology (Sangon, Shanghai, China), Lipofectamine 3000 (Invitrogen) was used to cotransfect according to the manufacturer’s instructions.

### pcDNA expression plasmids and RNA interference

The ORF sequences of TWIST, SNAIL, ZEB1, VEGFR1/2 and NRP1 were first amplified from the AGS cell line genomic DNA, and these ORF sequences were subcloned into the GV230 vector (GeneChem Corporation, Shanghai, China). Lipofectamine 2000 (Invitrogen) was used to transfect the plasmid into GC cells. After 1 month of transfection, AGS cell lines stably transfected with VEGFR1/2 and NRP1 were screened with 5 mg/ml G418 (Invitrogen, Cergy-Pontoise, France). The following siRNA sequences (Sangon Biotech, Shanghai, China) were transfected by Lipofectamine 2000 (Invitrogen) to silence the expression of SNAIL.

si-SNAIL sequences:

#1:F 5′-GCAGUUAAUUUAUAUAUUAAA-3′; R 5′-UAAUAUAUAAAUUAACUGCUU-3′

#2:F 5′-GUUUAUUGAUAUUCAAUAAAG-3′; R 5′-UUAUUGAAUAUCAAUAAACUG-3′

#3:F 5′-GCUUUGAGCUACAGGACAAAG-3′; R 5′-UUGUCCUGUAGCUCAAAGCAG-3′

(where not specified, siRNA #2 was used);

Control siRNAs (siC) sequence:

F 5′-UUCUCCGAACGUGUCACGUTT-3′, R 5′-ACGUGACACGUUCGGAGAATT-3′

### miRNA overexpression

The human miR-590 gene was cloned into a lentiviral vector produced by co-transfection of pHelper 1.0, pHelper 2.0 and plasmids pGC-LV into HEK293T cells by Lipofectamine 2000 (Invitrogen). Infection in the presence of 2 mg/mL polybrene (GeneChem Corporation), virus collection 48 h after transfection. GeneChem Corporation (Shanghai, China) provide the control miRNA viruses. After 15 days of infection, AGS cells stably infected with miR-590 were screened with 2 μg/μL puromycin (Invitrogen).

### Luciferase reporter assay

We constructed and inserted a 3 kb fragment upstream of the human miR-590 stem-loop with conserved Ebox motifs at −327 bp (E-box, CACATG); then, constructed and inserted a 3 kb fragment upstream of miR-590 that contained mutations of E-Box motifs. Next, we inserted wild-type or mutated fragments into the luciferase reporter plasmid PsicheckTM-2 (Sangon Biotech, Shanghai, China). Then, AGS cells were co-transfected with vectors overexpressing SNAIL or si-SNAIL. Finally, the luciferase activity was observed. The 3′UTR vectors of NRP1 and VEGFR1/2 containing a complete miR-590 recognition sequence were purchased from Geneseed Biotech Co. (Guangzhou, China). NRP1 and VEGFR1/2 each have one binding site in the 3′UTRs, so we designed primer sequences for the mutant 3′UTRs. The primer sequences were described below:

NRP1 forward: 5′-ATACTGCAACTTT-GATTAC-TAAAGTATCTTGCA-3′,

NRP1 reverse: 5′-TGCAAGATACTTTA-GTAATC-AAAGTTGCAGTAT-3′;

VEGFR1 forward: 5′- TAAAATAGCACTG-GATTAC-GAAACATGAATTAA-3′,

VEGFR1 reverse: 5′-TTAATTCATGTTTC-GTAATC-CAGTGCTATTTTA-3′;

VEGFR2 forward: 5′-TAGCCAGACTTC- GATTAC-ATTTTATAGCCCAAA-3′,

VEGFR2 reverse: 5′- TTTGGGCTATAAAAT-GTAATC-GAAGTCTGGCTA-3′;

In the luciferase assay, AGS cells were co-transfected with miR-590 and PsicheckTM-2 dual-luciferase vectors containing the WT or mutant target sequences by Lipofectamine 2000. 18 h after transfection, the dual-luciferase assay (Promega, Madison, WI, USA) was used to detect the activity of the firefly luciferase, the results were normalised according to Renilla luciferase activity. Transfection of each reporter plasmid and assay of each sample was repeated 3 times.

### ChIP assay

According to the manufacturer’s instructions, the ChIP-IT enzymatic kit (Active Motif, Carlsbad, CA) was used to perform the ChIP assay. After that, the specific antibodies (#sc-271977, Santa Cruz Biotechnology CA, USA) were used to immunoprecipitate Snail. Proteinase K was used to prepare the immunoprecipitated DNA and phenol/chloroform procedure was used for further purification. PCR primer sets as indicated:

Ebox: forward, 5′-TAGCACAAAGCATCAGTCCACA-3′,

Reverse: 5′-CTCTTCCTACCTTTCTGGGTTACA-3′;

Negative control: forward: 5′- AGAGGTTCCAGTGAGCCAAGA -3′,

Reverse: 5′- AAGCAAAACAAGTAAAGCGGTC -3′.

### Antibodies and immunoblotting

Antibodies against VEGFR1 (#ab32152), VEGFR2(#ab24313), NRP1(#ab81321), E-cadherin(#ab40772), N-cadherin(#ab76057), Snail(#ab229701), fibronectin(#ab32419), TWIST1(#ab50887), GAPDH(#ab181602), CD34(#ab81289), vimentin(#ab92547) and ZEB1(#ab203829) were purchased from Abcam (Cambridge, UK). An HRP-conjugated goat anti-rabbit IgG antibody (#sc-2004) was purchased from Santa Cruz Biotechnology. According to the manufacturer’s instructions, the total protein of GC tissues and transfected cells was extracted with RIPA lysis buffer (Beyotime, China). After the protein concentration was determined by the BCA protein assay, the equivalent amounts of cell lysates were electrophoresis with 10% SDS polyacrylamide gel and transferred onto polyvinylidene membrane, which was then placed on 5% skim milk prepared with TBS-T and sealed at 4 °C for 1 h. After that, the corresponding primary antibodies were used to incubate the blots. Incubation of blots with HRP-labelled secondary antibodies prior to visualise with enhanced chemiluminescence reagents (Millipore, Billerica, MA, USA).

### Immunofluorescence assay

Double immunostaining of VEGFR1/2, NRP1, N-cadherin, vimentin, fibronectin, E-cadherin and SNAIL at room temperature was performed as follows: 2.0 × 10^5^ AGS cells were seeded and grown on Nunc Thermanox coverslips (Thermo Scientific, NY, USA). After 24 h of culture, the cells were washed with 1× PBS and were fixed with 4% paraformaldehyde for 15 min. The protein that was expressed in the cytoplasm was infiltrated with 0.5% Triton-X100 PBS for 20 min. The coverslips were soaked in goat serum for 30 min. A sufficient amount of primary antibody was diluted and added according to the instructions on each coverslip and was placed in a wet box, which was incubated overnight at 4 °C. The next day, the coverslips were washed three times with PBS containing 0.5% Tween 20; subsequently, the coverslips were incubated for 1 h at room temperature in the wet box with a 1/1000-diluted secondary antibody (FITC). After the secondary antibody incubation, the coverslips were washed with PBS-T and were stained with 10 mM 4′,6′-dimidol-2-phenylhydrazine hydrochloride (DAPI) for 5 min. Finally, image acquisition was performed using an Olympus FV1000 confocal laser scanning microscope. The pictures were processed using ZEN software (Carl Zeiss Microscopy GmbH, Germany).

### Cell viability and clonability assays

According to the manufacturer’s instructions, the cell counting kit-8 (cck-8) system (Dojindo, Japan) was used to measure the activity of transfected cells inoculated into 96-well plates at a density of 1 × 104 cells/Wells. In short, before incubating the plate for 1 h at 37 °C in the dark, add 10 m of cck-8 solution to each well, and measure the absorbance of each well at 450 nm with the microplate reader (Tecan, Switzerland). In the colony formation assay, cells were seeded with low density (1000 cells/plate) and cultured until visible clones appeared, then stained with Giemsa and counted the number of colonies.

### Migration and invasion assays

In the transwell migration assays and the invasion assays, the cells were first placed in serum-free medium, and the medium supplemented with 10% serum was placed in the lower chamber as a chemoattractant. The former was seeded with 1 × 104 cells in an upper chamber with a non-coated membrane (24-well insert; 8 mm pore size; BD Biosciences), and the latter seeded with 2 × 105 cells with a Matrigel-coated membrane (24-well insert; 8 mm pore size; BD Biosciences). The cells were incubated in a tissue culture incubator at 37 °C and 5% CO 2 for 16 h, after which the ummigrated/non-invasive cells on the upper sides of the Transwell membrane filter insert were gently wiped off with a cotton swab. On the underside of the insert, cells were stained with crystal violet and counted.

### Animal studies

All animal experiments were performed following protocols of Guide for the Care and Use of Laboratory Animals of Tongji Hospital at Tongji Medical College, which were approved by the Committee on the Ethics of Animal Experiments of Tongji Hospital at Tongji Medical College. To minimise the pain, all operations were performed under sodium pentobarbital anaesthesia. Male nude mice aged 6–8 weeks in the Animal Experimental Center of Tongji Hospital of Tongji Medical College started the experiment after two weeks of adaptation. During the experiment, the investigator was blinded to the group allocation. Male nude mice were randomly divided into six groups (*n* = 5). The posterior side of each mouse was injected subcutaneously with 100 μl of PBS solution containing equal amounts of AGS cells overexpressing miR-590 and with or without VEGFR1/2 or NRP1 restoration. The tumour growth of each group was observed every day. The tumour volume was calculated as: *V* = 1/2 a × b2, where a is the longest axis of the tumour and b is the shortest. During the animal experiments, if nude mice die or those xenograft tumour don’t grow, we will exclude those mice out of experiments. After 5 weeks, the mice were sacrificed and tumours were removed. A part of the tumours were fixed in formalin and subjected to immunohistochemical analysis, and the other part was frozen in liquid nitrogen and subjected to qRT-PCR.

Each 6–8-week-old male nude mice were randomly into six groups (*n* = 5). The lateral tail veins of mice were injected with AGS cells (10^6^ cells in 200 μL PBS). The metastasis of each mouse was detected by the IVIS system. The mice were sacrificed on day 30, the mammary tumours, mouse liver and lungs were taken off and fixed in formalin and then histologically examined by paraffin embedding.

### Immunohistochemistry

Paraffin-embedded tumour sections were placed on adherent slides, then deparaffinized in xylene, and further dehydrated with different concentrations of alcohol, and then pretreated in citrate buffer for 20 min in a 98 °C steamer, for antigen retrieval. Sections were incubated overnight at 4 °C with antibodies against CD34, NRP1 or VEGFR1/2. The UltraSensitive S-P Detection Kit (KIT-9720, Maixin, Fuzhou, China) was used to perform immunostaining, and the DAB kit (PW017, Sangon Biotech, Shanghai, China) was used to develop the colour. Subsequently, the haematoxylin was used to counterstain sections. Morphological assessment was finally performed using H&E staining.

### Immunohistochemical stain intensity quantification

Randomly selected five brightfield microscopy images (magnification 40 times; 0.89 mm^2^) per sample obtained as described above, then counted positive microvessels with ImageJ program to confirm tumour microvessel density.The red channel corresponding to CD34 staining was separated and digitised into a binary image, black for staining blood vessels and white for unstained blood vessels. The lumens of the vessels were digitally filled and the D-MVA was quantitatively synthesised. The DAB-stained brown-coloured images were extracted by a colour deconvolution macro using the ImageJ program. After averaging the intensity values of the each group, the ratios between the groups were calculated.

### Statistical analysis

All data were expressed as mean ± standard deviation (s.d.), Student’s *t* test was used for comparison between groups, and all statistical analyses were performed using SPSS 17.0 software. The Kaplan–Meier (log-rank test) was used to analyse disease progression. The relationship among the relative expression levels of miR-590 and VEGFR1/2, NRP1 and D-MVA in GC tissues was evaluated by the Spearman’s rank test. Differences were considered significant if *p* < 0.05.

## Results

### miRNA selection

**Fig. 1:**
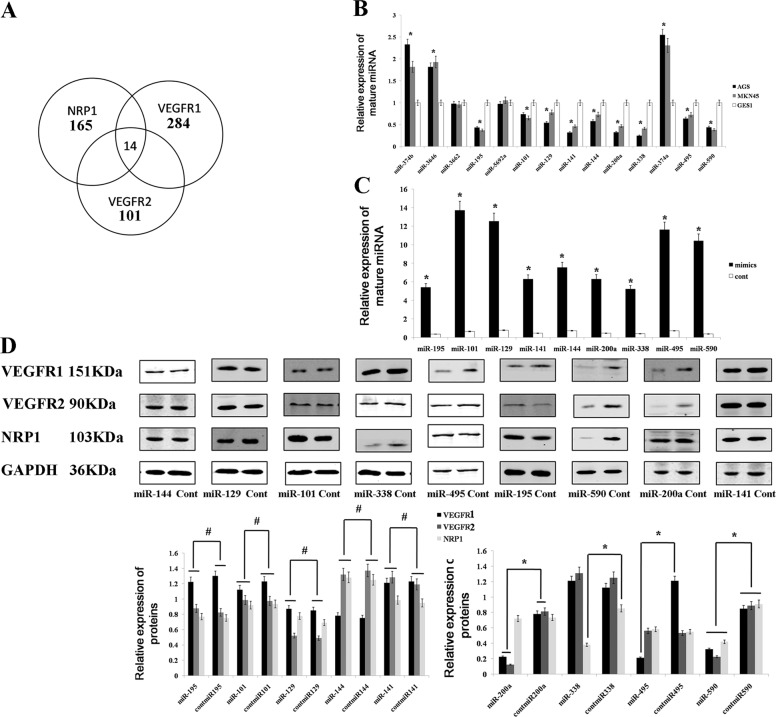
miRNAs were selected by prediction tools and a western blot analysis. **a** We selected 14 miRNAs that simultaneously curbed the expression of VEGFR1/2 and NRP1 using a Venn diagram. **b** The expression of miRNAs was detected in gastric cancer lines (AGS and MKN45) and in a normal gastric mucosal cell line (GES1) by qRT-PCR analysis. **c** The miRNA expression was significantly increased in the gastric cancer cells after transfection with miRNA mimics. **d** VEGFR1/2 and NRP1 expression was examined by a western blot in AGS gastric cell lines after transfecting miR-590 mimics and cont-miR. The data are shown as the mean ± s.d. (*n* = 3) in cell lines *p < 0.05.

According to the prediction tools PicTar (http://pictar.bio.nyu.edu/), Miranda (http://www.microrna.org), TargetScan (http://genes.mit.edu/targetscan/index.html) and miRDB (mirdb.org/miRDB/), we predicted some miRNAs that may regulate the expression of VEGFR1/2 or NRP1 (Supplemental Table 1). Then, a Venn diagram was used to identify the miRNAs that can simultaneously curb the expression of VEGFR1/2 and NRP1 (Fig. 1a). The expression of those miRNAs was examined by qRT-PCR, and we found that the expression of miR-195 and miR-101 was downregulated in GC (Fig. 1b). To further investigate whether those overexpressed miRNAs could inhibit the expression of NRP1 and VEGFR1/2, we upregulated the expression of those miRNAs in the GC cell line AGS (Fig. 1c), and we found that overexpressed miR-338 could inhibit the expression of NRP1. Overexpressed miR-459 could inhibit VEGFR1, and only overexpressed miR-590 could inhibit the expression of both VEGFR1/2 and NRP1 (Fig. 1d).

### miR-590 directly targets VEGFR1/2 and NRP1

**Fig. 2:**
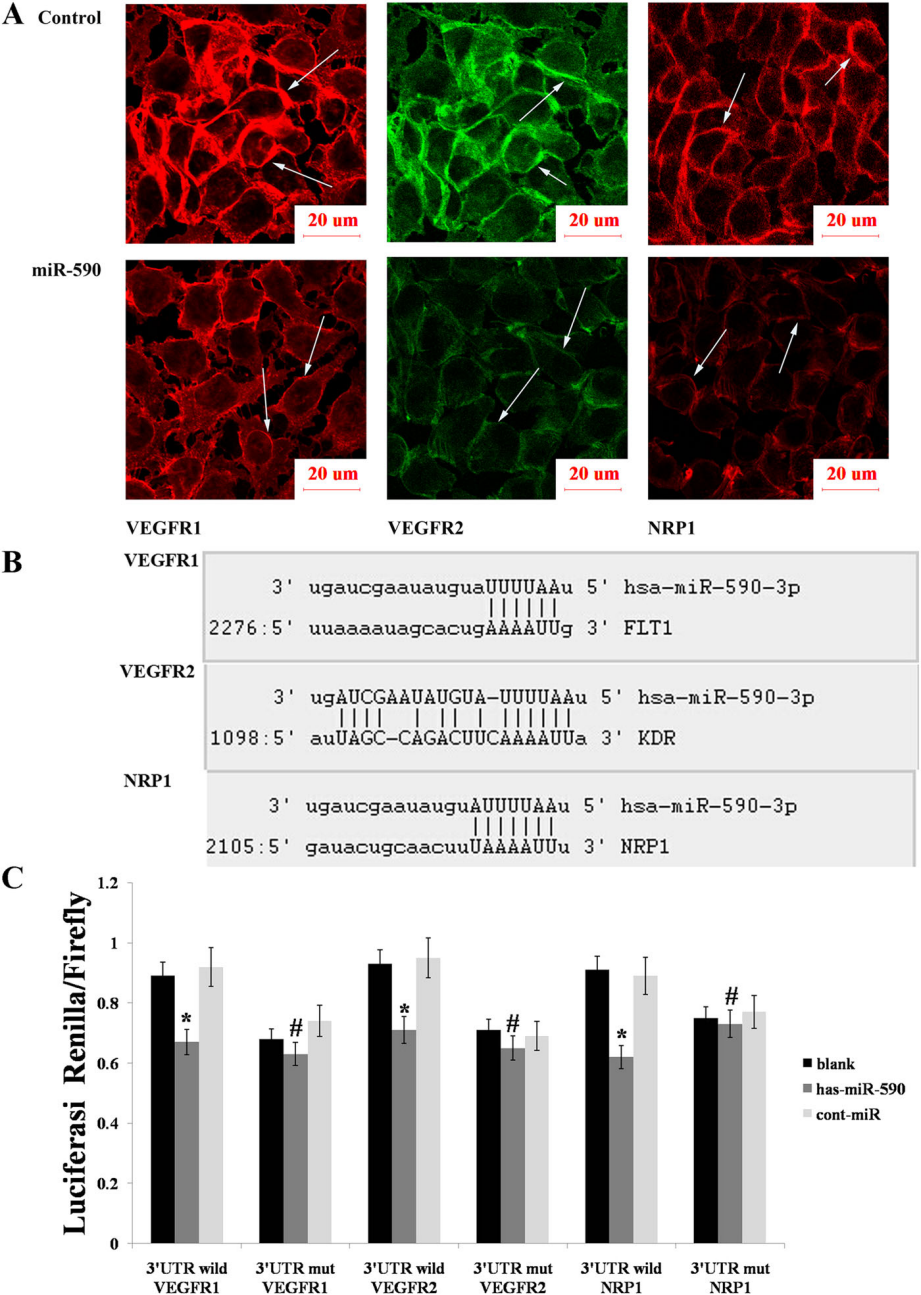
miR-590 decreased the VEGFR1/2 and NRP1 expression levels by directly targeting the 3′UTRs. **a** The fluorescence intensity of VEGFR1/2 and NRP1 was significantly reduced after transfection with the miR-590 mimics. The arrows indicate VEGFR1/2 or NRP1. **b** The sequences of the predicted miR-590 binding sites within the VEGFR1/2 or NRP1 3′UTRs are shown. **c** The relative luciferase activity was analysed after the reporter plasmids were cotransfected with the miR-590 mimics or the control mimics into AGS cell lines. The data are shown as the mean ± s.d. (*n* = 3) in cell lines *p < 0.05, ^#^*p* > 0.05.

By using immunofluorescence, we also demonstrated that miR-590 could curb the expression of both VEGFR1/2 and NRP1 (Fig. 2a). Luciferase reporter assay was performed to confirm whether miR-590 can directly targets VEGFR1/2 and NRP1, and it revealed the presence of a potential binding site for miR-590 in the 3′UTRs of VEGFR1/2 and NRP1 (Fig. 2b). We then constructed a binding site mutant and a full-length wild-type VEGFR1/2 or NRP1 3′utr to verify whether miR-590 regulates the expression of VEGFR1/2 or NRP1 by binding directly to their 3′UTRs. Insertion of the above fragment into the psiCHECK2 luciferase reporter plasmid revealed that co-transfection of miR-590 and wild-type VEGFR1/2 or NRP1 3′UTR resulted in a significant decrease in luciferase activity compared with control groups, whereas miR-590 and mutation co-transfection of the 3′UTR did not result in a significant change in luciferase activity (Fig. 2c).

### miR-590 is downregulated in gastric cancer cell lines and tissues

**Fig. 3:**
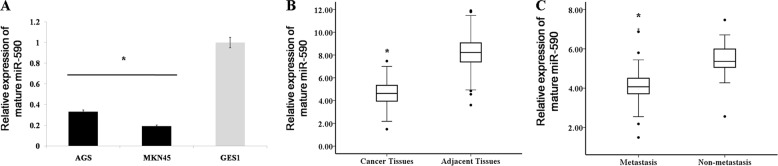
miR-590 was downregulated in gastric cancer tissues and cell lines. **a** miR-590 expression was detected by qRT-PCR analysis in gastric cancer cell lines (AGS, MKN45) and in a normal gastric mucosa cell line, GES1. The data are shown as the mean ± s.d. (*n* = 3) in cell lines, *p < 0.05. **b** The expression levels of mature miR-590 in gastric cancer (*n* = 118) and in adjacent normal mucosal tissues (*n* = 118) were determined by qRT-PCR analysis. The data are shown separately in human samples; * *p* < 0.05. **c** Mature miR-590 expression levels in metastatic (*n* = 72) and non-metastatic (*n* = 46) gastric cancers are shown. The data are shown separately in human samples; *p < 0.05.

By detecting the expression of miR-590 in several human cancer cell lines (AGS and MKN45) and normal gastric mucosal cell line (GES1), we found that the expression level of miR-590 in GC cells was lower than normal (Fig. 3a). Subsequently, we examined the expression levels of miR-590 in 118 human GC specimens and corresponding adjacent normal mucosa specimens to investigate the role of miR-590 in human GC. The clinical data are shown in Table 1. QRT-PCR results showed that the expression level of miR-590 in tumour tissues was significantly decreased than that in adjacent normal mucosa tissues (Fig. 3b). To confirm the relationship between miR-590 expression and GC metastasis, we divided primary gastric tumours into lymph node metastasis and lymph node metastasis. QRT-PCR results showed that the expression level of miR-590 in the metastatic group was significantly decreased than that in the non-metastasis group. (Fig. 3c).

**Table 1:** Clinical characteristics of the patient cohort.

Characteristics	Number of patients	Expression of miR-590 in gastric cancer	*P* values
Age (years)			
≤60	58	4.70 ± 0.97	0.544
>60	60	4.58 ± 1.11	
Gender			
Male	67	4.70 ± 0.95	0.528
Female	51	4.58 ± 1.12	
Smoke			
Yes	62	4.59 ± 1.13	0.645
No	56	4.68 ± 0.94	
Drinking History			
No	56	4.72 ± 0.96	0.373
Yes	62	4.55 ± 1.12	
HP infection			
Yes	86	4.54 ± 1.10	0.123
No	32	4.87 ± 0.84	
Pathological T			
PT0-T2	55	4.80 ± 1.05	0.12
PT3-T4	63	4.49 ± 1.03	
Tumour Differentiation			
Poorly	43	4.57 ± 1.09	0.398
High/Middle	75	4.74 ± 0.97	
Lymph Node Metastasis			
N0	46	5.43 ± 0.74	0.001*
N1–3	72	4.12 ± 0.88	

The bold value in Table 1 indicates that there is significant difference between metastasis group and non-metastasis group.

### The correlations and prognostic significance of miR-590 in gastric cancer

**Fig. 4:**
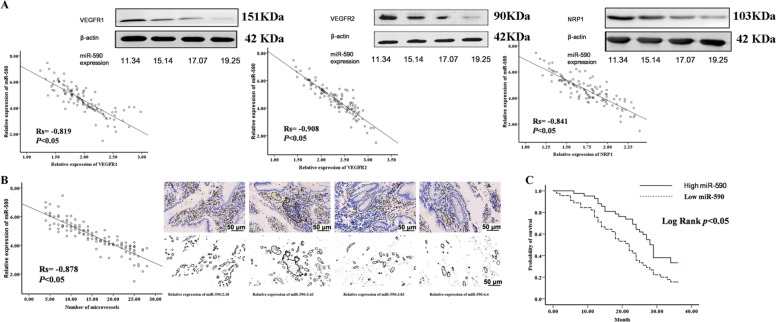
The correlations and prognostic significance of miR-590 were shown in gastric cancer. **a** VEGFR1/2 or NRP1 protein expression was examined by western blot in gastric cancer tissues, and the correlation between miR-590 expression and VEGFR1/2 or NRP1 protein expression is shown. **b** Binary images of CD34 staining with the lumen digitally filled are shown, and the correlation between miR-590 expression and D-MVA in gastric cancer tissues is also shown. **c** Kaplan–Meier analysis of overall survival based on miR-590 expression. The data are shown separately in human samples; *p < 0.05.

Western blot was used to detect the expression levels of VEGFR1/2 and NRP1 in GC tissues. It was found that the expression of miR-590 was inversely correlated with VEGFR1/2 and NRP1 (Fig. 4a). The number and morphological characteristics of blood vessels in each tumour were assessed by immunohistochemical staining of CD34. Meanwhile, we incorporated the size and patency of blood vessels into our tumour vascular system analysis to analyse the digital microvascular region (D-MVA) by estimating the integrated lumen region and assuming vertical blood flow to the tumour section. We also found that the expression of miR-590 was inversely correlated with D-MVA (Fig. 4b). GC cases were divided into low miR-590 group and high miR-590 group (by median) according to the expression level of miR-590, and the Kaplan–Meier survival analysis was performed to determine whether the levels of miR-590 in tumour tissues were related to the survival of patients with GC. According to the Kaplan–Meier analysis, the high miR-590 group had significantly improved overall survival relative to the survival of the low miR-590 group (Fig. 4c). Unfortunately, no significant correlation was observed between the expression of VEGFR1/2 and NRP1 and GC overall survival (Supplemental Fig. 1).

### miR-590 is directly repressed by the transcription factor SNAIL

**Fig. 5:**
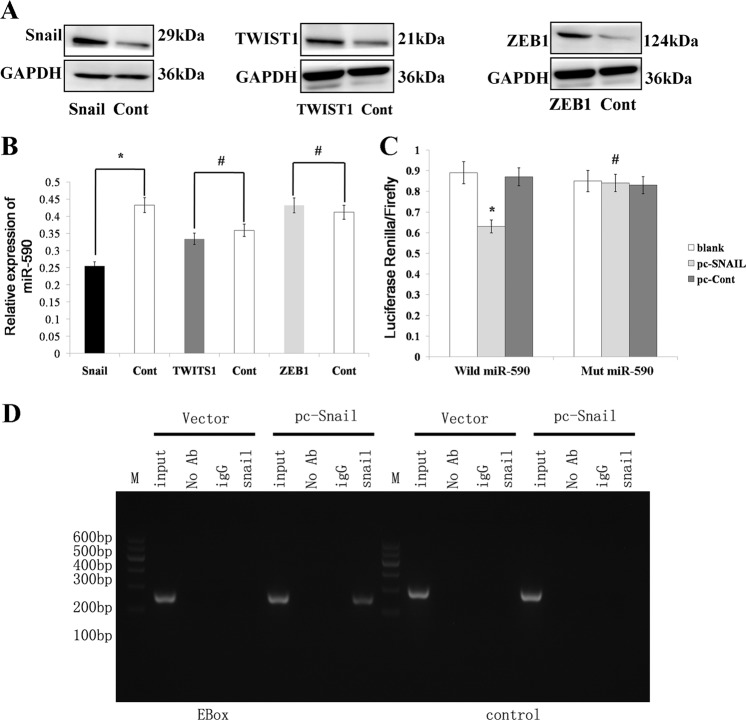
miR-590 is directly repressed by SNAIL. **a** The expression of TWIST, SNAIL and ZEB1 was examined by a western blot after the SNAIL, TWIST and ZEB1-expressing vector was transfected into the AGS cell lines. **b** The expression of miR-590 was detected by qRT-PCR after overexpressing SNAIL, TWIST and ZEB1. **c** The SNAIL-expressing vector was cotransfected with the psiCHECK2 luciferase reporter plasmid, which contained a 3 kb fragment upstream of the human miR-590 stem-loop, into AGS cells. The luciferase activity was then observed. **d** A ChIP assay analysis was performed in AGS cells transfected with a vector expressing SNAIL. The data are shown as the mean ± s.d. (*n* = 3) in cell lines *p < 0.05, ^#^*p* > 0.05.

Peinado H et al.^22^ found that bHLH, ZEB and the SNAIL families can bind to E-box sequences in the promoters. We also proved that SNAIL can bind to E-box sequences in the promoters of miR-128^23^ in our previous study. We used rVista 2.0^24^ to search for conserved transcription factor binding sites and identified conserved Ebox motifs relative to the human miR-590 stem-loop transcription initiation site (+1)−327 bp (E-box, CACATG). We constructed the TWIST (bHLH family), ZEB1 (ZEB family) and SNAIL (snail family) expression vectors (Fig. 5a) to detect transcription factors that regulate miR-590 expression; we then examined the expression of miR-590 after the overexpression of TWIST, SNAIL or ZEB1. We found that the expression of miR-590 was downregulated after the overexpression of SNAIL compared with that in the control group, but the expression of miR-590 was not regulated after the overexpression of TWIST and ZEB1 (Fig. 5b). SNAIL expression vector containing a 3 kb fragment upstream of the miR-590 stem-loop was cotransfected into AGS cells, and SNAIL significantly inhibited the relative luciferase activity of miR-590; however, mutations of the E-Box motif abrogated the responsiveness to SNAIL (Fig. 5c). To further determine the relationship between SNAIL and miR-590, we knocked down the expression of SNAIL by RNA interference (RNAi) (Supplemental Fig. 2). Conversely, the activity of the luciferase construct of miR-590 was increased after depletion of SNAIL with a specific siRNA, while the sites mutation of E-Box eliminate this increase (Supplementary Fig. 3). ChIP assays were used to investigate whether miR-590 was directly regulated by SNAIL, the results indicate that the SNAIL bound to the E-box but did not bind to the E-box in the negative control (Fig. 5d).

### miR-590 inhibits gastric cancer cell migration, invasion and proliferation by VEGFR1/2 and NRP1

**Fig. 6:**
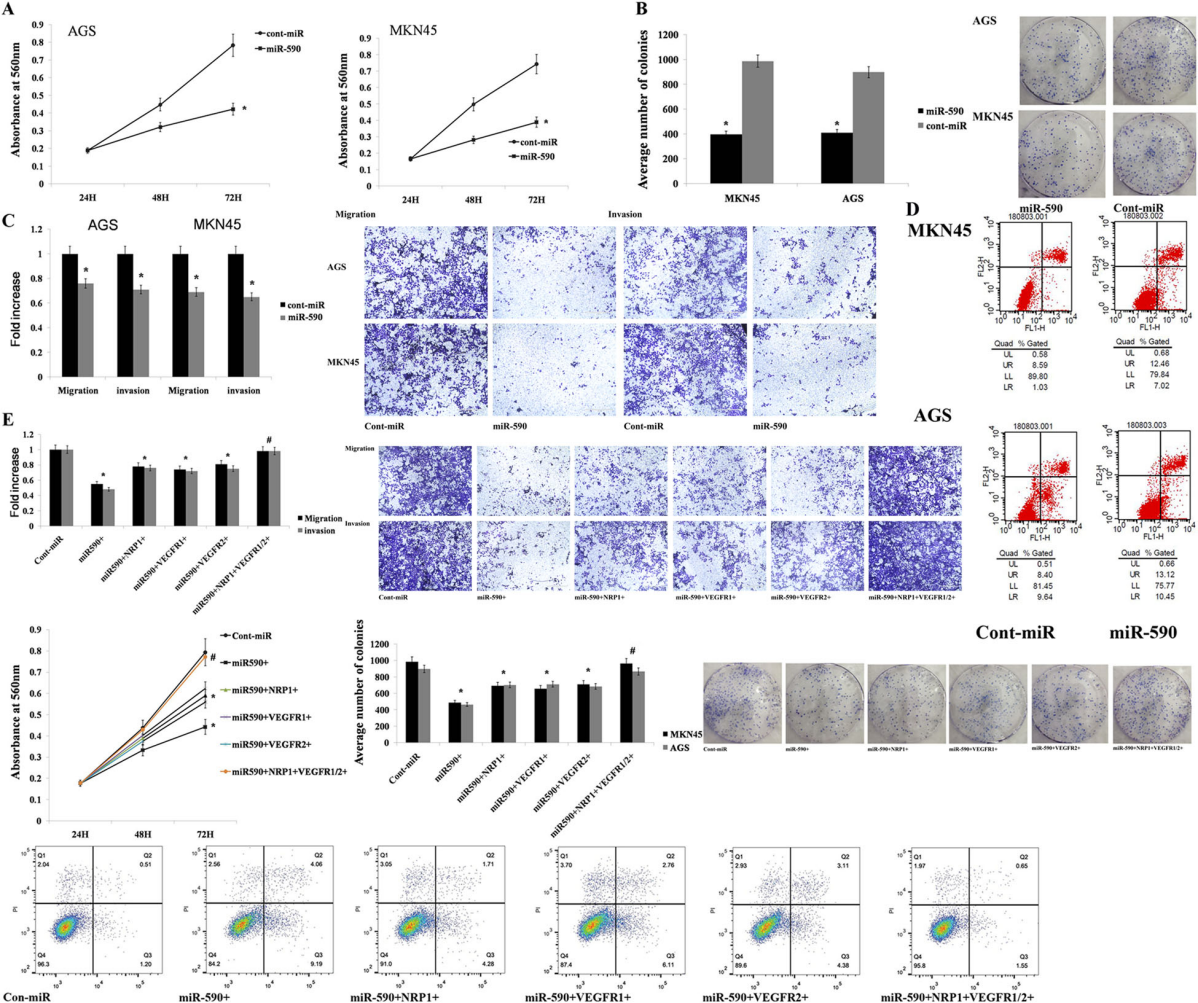
miR-590 inhibits gastric cancer cell migration, invasion and proliferation and promotes apoptosis. **a** Gastric cancer cell proliferation was significantly reduced after miR-590 mimic transfection compared with that after cont-miR transfection. **b** miR-590 overexpression significantly inhibited the colony-forming ability of gastric cancer cells. **c** miR-590 overexpression significantly reduced the migration and invasion capacities of AGS and MKN45 cells compared with the capacities of the controls. **d** miR-590 overexpression significantly increased gastric cancer cell apoptosis. **e** Gastric cancer cell migration, invasion, proliferation and apoptosis were partially restored after VEGFR1/2 or NRP1 restoration and were completely restored after overexpressing both VEGFR1/2 and NRP1. The data are shown as the mean ± s.d. (*n* = 3) in cell lines *p < 0.05.

We transduced miR-590 mimics into AGS and MKN45 GC cell lines to investigate the functional significance of miR-590 in GC. We found that the level of proliferation of cells overexpressing miR-590 was significantly decreased compared with the control group (Fig. 6a). Overexpression of miR-590 cells showed a decrease in colony-forming ability, and the number of foci was also significantly reduced compared with the control group (Fig. 6b). The Transwell migration and Matrigel invasion assays were performed to demonstrate that the migration and invasion of AGS and MKN45 cells were significantly reduced by miR-590 (Fig. 6c). Overexpression of miR-590 in AGS and MKN45 cells resulted in a significant increase in the percentage of total apoptotic cells (early apoptosis + late apoptosis) (Fig. 6d).

We overexpressed miR-590 in AGS cell lines together with a construct containing the VEGFR1/2 or NRP1 coding sequence but lacking the mRNA 3′UTR, thereby further confirming whether miR-590 promotes GC migration, invasion and proliferation by targeting VEGFR1/2 and NRP1. Therefore, this construct yielded a VEGFR1/2 or NRP1 mRNA that was resistant to miR-590. Overexpression of VEGFR1/2 or NRP1 partially restored migration, invasion and proliferation of GC cells, and these functions were fully restored after simultaneous overexpression of VEGFR1/2 and NRP1 (Fig. 6e).

### miR-590 induces the reversion of the EMT via VEGFR1/2 and NRP1

**Fig. 7:**
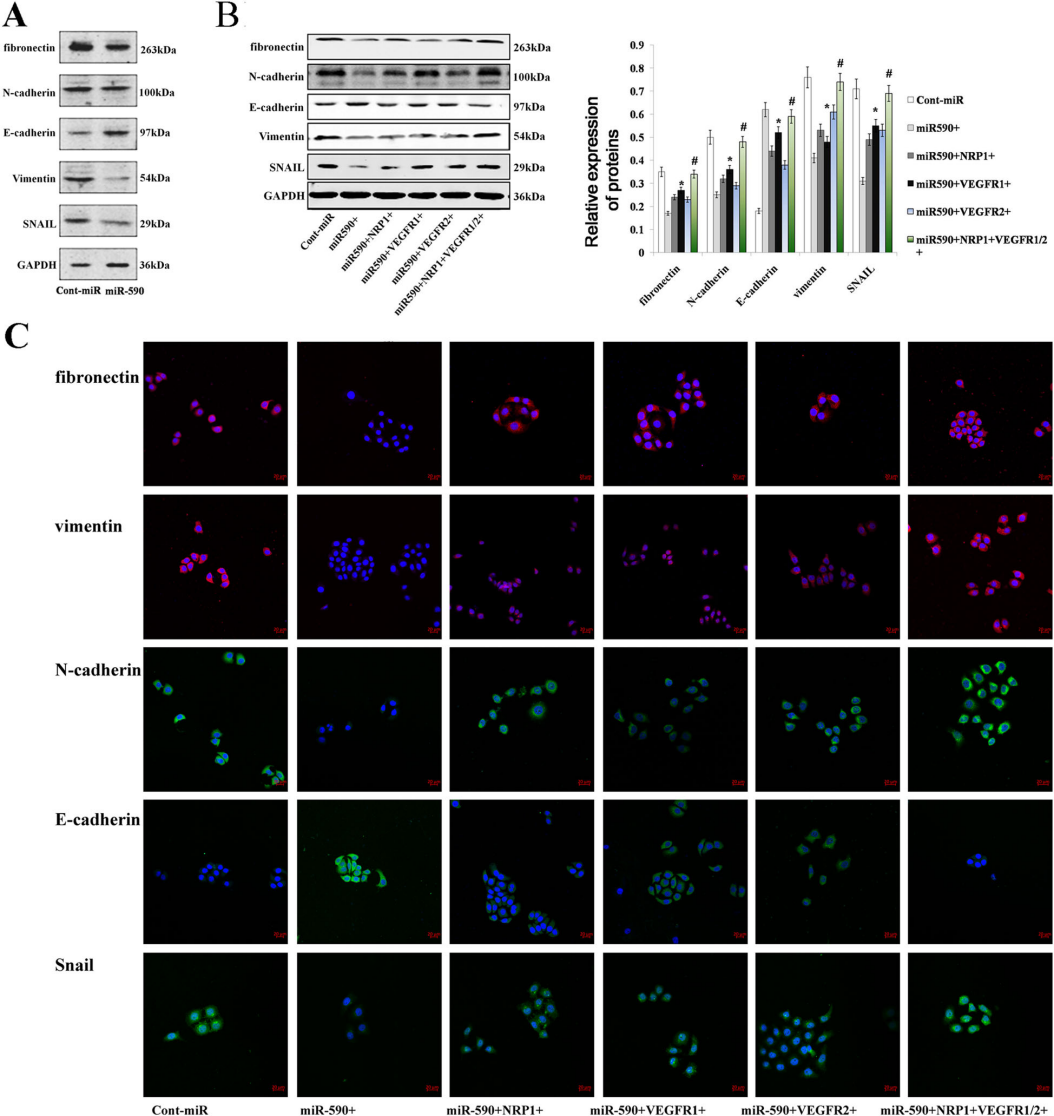
miR-590 promotes an epithelial phenotype in gastric cancer. **a** An immunoblot analysis of N-cadherin, vimentin, fibronectin, E-cadherin and SNAIL in AGS cells transfected with miR-590 mimics or cont-miR. **b** An immunoblot analysis of N-cadherin, vimentin, fibronectin, E-cadherin and SNAIL in AGS cells transfected with miR-590 mimics or cont-miR with or without VEGFR1/2 or NRP1 restoration. The protein expression levels were normalised to GAPDH. **c** The fluorescence intensity of N-cadherin, vimentin, fibronectin, E-cadherin and SNAIL was examined by confocal microscopy after transfection with miR-590 mimics or cont-miR with or without VEGFR1/2 or NRP1 restoration. The data are shown as the mean ± s.d. (*n* = 3) in cell lines *p < 0.05.

EMT (epithelial-mesenchymal transition) is characterised by the gain of a mesenchymal phenotype with the expression of mesenchymal proteins (including N-cadherin, vimentin, fibronectin and SNAIL) and the loss of epithelial cell markers (such as E-cadherin)^25^. The EMT is an important mechanism that is associated with cancer invasiveness and metastasis. In our previous study, we demonstrated that miR-338 could inhibit the EMT via NRP1 in GC cells^26^. We also knew that VEGFR1/2 might contribute to the EMT in certain types of cancers^27,28^. Because miR-590 could mitigate the expression of VEGFR1/2 and NRP1 in GC, we inferred that miR-590 could determine the epithelial phenotype of GC. The immunoblot analysis showed that overexpression of miR-590 in AGS cells caused a decrease in the expression levels of fibronectin, vimentin, N-cadherin and SNAIL, and increased expression of E-cadherin (Fig. 7a). Rescue experiments were also performed to confirm whether miR-590 could regulate the EMT via VEGFR1/2 and NRP1. We used immunoblot analysis to demonstrate that the above mesenchymal marker expression partially restored to the normal level after VEGFR1/2 or NRP1 was restored in the miR-590-expressing cells, and the expression of the mesenchymal markers was completely restored to the normal level after overexpressing both VEGFR1/2 and NRP1 (Fig. 7b). These results were also confirmed by immunofluorescence (Fig. 7c).

### miR-590 decreases tumour growth and metastasis by targeting VEGFR1/2 and NRP1 in vivo

**Fig. 8:**
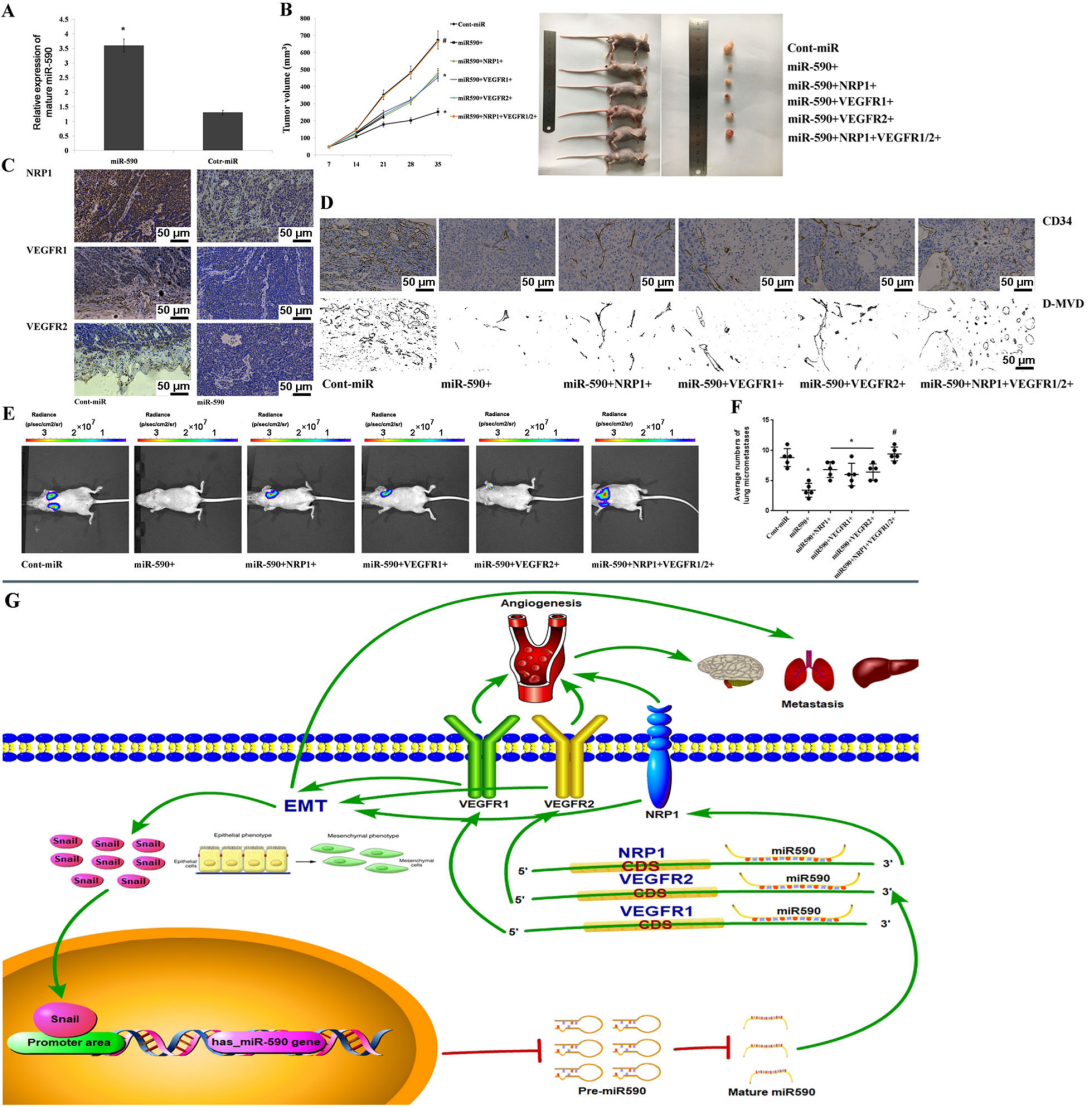
miR-590 decreases tumour growth and metastasis by VEGFR1/2 and NRP1 in nude mice. **a** miR-590 expression in tumour xenografts was measured using qRT-PCR after transfection with miR590 or cont-miR. **b** Left panel: Primary tumour growth from nude mice with forced expression of miR-590 or cont-miR, with or without VEGFR1/2 and NRP1 restoration. Right panel: Representative image of xenograft tumours are shown. **c** VEGFR1/2 or NRP1 expression in tumour xenografts was detected by immunohistochemical staining. **d** D-MVD in tumour xenografts was detected by immunohistochemical staining with the forced expression of miR-590 or cont-miR, with or without VEGFR1/2 and NRP1 restoration. **e** Representative IVIS images of lung metastases in nude mice that received a tail vein injection of miR-590 or cont-miR with or without VEGFR1/2 or NRP1-infected AGS cells are shown. **f** The average numbers of lung micrometastases in nude mice. The data are shown separately in nude mice; *p < 0.05. **g** Schematic representation of Snail-miR590-VEGFR-NRP1 axis. Green arrow means activation, and red arrow means inhibition.

Transfection of miR-590 in AGS and MKN45 can cause a decrease in cell migration, invasion and proliferation, so we wonder whether miR-590 has a similar effect in the growth of GC in vivo. Equal numbers (1 × 10^6^ cells per mouse) of stable AGS cells overexpressing miR-590 or cont-miR with or without VEGFR1/2 or NRP1 restoration were injected subcutaneously into nude mice. QRT-PCR demonstrated a significant increase in the expression level of miR-590 in tumour xenografts overexpressing miR-590 (Fig. 8a). By measuring the volume of tumour xenografts we found that the tumour growth in vivo could significantly inhibited by overexpressing miR-590, while overexpression of VEGFR1/2 or NRP1 partially restored tumour growth, and the overexpression of both VEGFR1/2 and NRP1 completely restored the tumour growth (Fig. 8b). Next, we examined VEGFR1/2 and NRP1 expression and D-MVD in tumour xenografts by immunohistochemistry. We found that VEGFR1/2 and NRP1 expression significantly decreased in the tumours that overexpressed miR-590 (Fig. 8c). D-MVD also decreased in the tumour xenografts that overexpressed miR-590; D-MVD was partially restored to the normal level after overexpressing VEGFR1/2 or NRP1, and D-MVD was completely restored to the normal level after overexpressing both VEGFR1/2 and NRP1 (Fig. 8d). To investigate the effect of miR-590 on the metastasis of GC in vivo, stable AGS cells overexpressing miR-590, with or without VEGFR1/2 or NRP1 restoration, were injected into the tail vein of nude mice. After 30 days, we detected metastases by Caliper IVIS Lumina II. Lung metastasis in nude mice injected with miR-590 forced expressing AGS cells was less than that in the control group, overexpression of VEGFR1/2 or NRP1 partially restored lung metastases, and the overexpression of both VEGFR1/2 and NRP1 completely restored lung metastases (Fig. 8e, f). Combination with in vitro and in vivo experimental results, we can conclude that miR-590 inhibits GC growth and metastasis via tumour angiogenesis and EMT pathway by targeting VEGFR1/2 and NRP1. Meanwhile, Snail, as a elevated EMT cell marker, also can inhibit the expression of miR-590 with a positive feedback mechanism (Fig. 8g).

## Discussion

Recent studies have shown that VEGFR-1 and VEGFR-2 are expressed in many cancers and are closely related to tumour angiogenesis. Chen H et al.^29^ believe that VEGFR2 is closely related to vascular density and tumour progression in NSCLC. Alip Ghosh et al.^30^ found that VEGFR1/2 promotes the growth, invasion and angiogenesis of liver cancer. As a co-receptor of VEGFR, NRP1 also plays a key role in angiogenesis, development, tumour growth and metastasis^14^. There have been many studies on the upstream regulation mechanism of VEGFR 1/2 or NRP1, but whether VEGFR 1/2 and NRP1 have a common upstream regulation mechanism in GC is still unclear. This is the main reason for us to explore the possible miRNAs that simultaneously regulate VEGFR 1/2 and NRP1. The prediction tools predicted that miR-590 might inhibit both VEGFR 1/2 and NRP1, and further experiments proved that VEGFR 1/2 and NRP1 were direct targets of miR-590.

miR-590 was aberrantly expressed in various cancers. In lung adenocarcinoma^31^ and cervical cancer^32^, miR-590 is upregulated, while in breast cancer^33^, miR-590 is downregulated. First, we detected the expression of miR-590 by qRT-PCR in GC cell lines, and we found that the expression of miR-590 was significantly decreased compared with that of GES1. Thus, miR-590 may act as an anti-oncogene in GC. Second, to further discuss the function of miR-590 in metastasis, we detected the expression of miR-590 in GC with or without lymph node metastasis, and we found that the expression of miR-590 in GC tissues with lymph node metastasis was significantly lower than that in those without metastasis. The results showed that miR-590 may have some roles in GC metastasis. As miR-590 was the upstream gene of VEGFR 1/2 and NRP1, which could regulate the expression of angiogenesis in GC, then the expression of VEGFR 1/2 and NRP1 and the microvessel density was examined in GC. It was found that the expression of VEGFR 1/2 and NRP1 and the microvessel density in GC tissues were negatively correlated with miR-590. Thus, miR-590 could mitigate GC angiogenesis by curbing the expression of VEGFR 1/2 and NRP1. Because vascular invasion was the primary cause of GC patient death, the results also explain why miR-590 was negatively correlated with all causes of mortality in GC patients.

In fact, the reason for the downregulation of miR-590 in GC is not clear. Studies have shown that miRNA is regulated by transcription factors^34^. PengXu Q et al. found that the transcription factor Snail could bind to e-box1 and e-box2 in the promoter region of mir-128, inhibiting the expression of mir-128^35^. Using the prediction tool we found that if the transcription initiation site of miR-590 stem-loop is (+1), the conserved Ebox motif is at −327 bp (E-box, CACATG). Therefore, we speculate that miR-590 may also be regulated by transcription factors in GC. Through ChIP and luciferase reporter assays, we found that SNAIL can directly bind to the e-box in the promoter region of miR-590. Meanwhile, the results also showed that the expression of miR-590 was also downregulated by SNAIL. SNAIL is a zinc finger transcriptional repressor composed of SNAI1 (SNAIL), SNAI2 (SLUG) and SNAI3 (SMUC). It has been demonstrated that the targets of SNAI1 are relevant to tumour development^36^ and tumour recurrence^37^. SNAIL can regulate the proliferation, invasion and apoptosis of various cancers by regulating miRNA has been shown in many studies. Xu et al. found that miR-375 may be negatively regulated by Snail and may be involved in GC cell migration and invasion^38^. According to our results, we found that the miR-590 expression was downregulated by SNAIL, and we speculate that the low miR-590 expression may be related to the occurrence and development of GC.

To further determin the functions and effects of miR-590, the plate cloning assay and the Transwell migration assay were performed to test the effects of miR-590 on the growth and invasion of GC cells. The results showed that the overexpression of miR-590 could significantly inhibit the growth, proliferation, migration and invasion of GC cell lines and could promote apoptosis. Moreover, only the overexpression of VEGFR1/2 and NRP1 can fully restore the above changes. In orthotopic implantation, the forced expression of miR-590 significantly inhibited tumour growth, and the forced expression of VEGFR1/2 and NRP1 restored the tumour growth. In addition, in the metastatic nude mouse model, the overexpression of miR-590 significantly reduced the proportion of lung metastasis, and the overexpression of VEGFR1/2 and NRP1 restored the metastatic tendency. In summary, our results show that miR-590 can inhibit the migration, invasion, proliferation and metastasis of GC in vivo and in vitro by targeting VEGFR1/2 and NRP1. GC metastasis, which is closely related to EMT, is the leading cause of death in patients. Many studies have found that VEGFR1/2 and NRP1 can regulate the EMT. Luo et al.^39^ found that VEGF or NRP-1 silencing attenuated the EMT in breast cancer. Therefore, we speculate that miR-590, which can simultaneously regulate VEGFR1/2 and NRP1 in GC, can regulate the occurrence of EMT. In our study, western bolt and immunofluorescence assays confirmed that miR-590 inhibits the EMT of GC cells by targeting VEGFR1/2 and NRP1. Our results demongtrate that SNAIL is a transcriptional repressor that directly regulates the expression of miR-590 in GC. We also found that miR-590 can target both VEGFR1/2 and NRP1 to regulate the expression of the mesenchymal marker SNAIL. Here, we find a new possible positive feedback loop. In GC cells, due to the presence of the transcription factor SNAIL, the expression of miR-590 is decreased; this causes the upregulation of the expression of NRP1 and VEGFR1/2, which leads to the occurrence of EMT in GC. Essentially, the expression of Snail increases. Overexpressed SNAIL then acts again on the upstream promoter of miR-590, inducing a decrease in miR-590 expression. These results reveal a new cascade of reactions in the oncogenic transformation of GC.

In summary, our results show that miR-590 is downregulated in GC, and the downregulation is due to the presence of the transcription factor SNAIL. In addition, miR-590 can inhibit the growth, migration, invasion, proliferation and metastasis of GC in vivo and in vitro by targeting VEGFR1/2 and NRP1. Therefore, miR-590 is a potential biomarker for the prognosis of GC. Finally, miR-590 inhibits the EMT by targeting VEGFR1/2 and NRP1. The understanding of the snail/miR-590 axis provides new insights into the carcinogenic transformation mechanisms of GC.

## Supplementary information

supplement Fig1

supplement Fig2

supplement Fig3

Supplement Tab. 1

Supplementary legends
