# Supplementary figures and images for: The regulatory mechanism and biological significance of the Snail-miR590-VEGFR-NRP1 axis in the angiogenesis, growth and metastasis of gastric cancer
Source: Cell Death Dis. 2020 Apr 17;11(4):241. doi: 10.1038/s41419-020-2428-x (PMC7165172; doi:10.1038/s41419-020-2428-x)

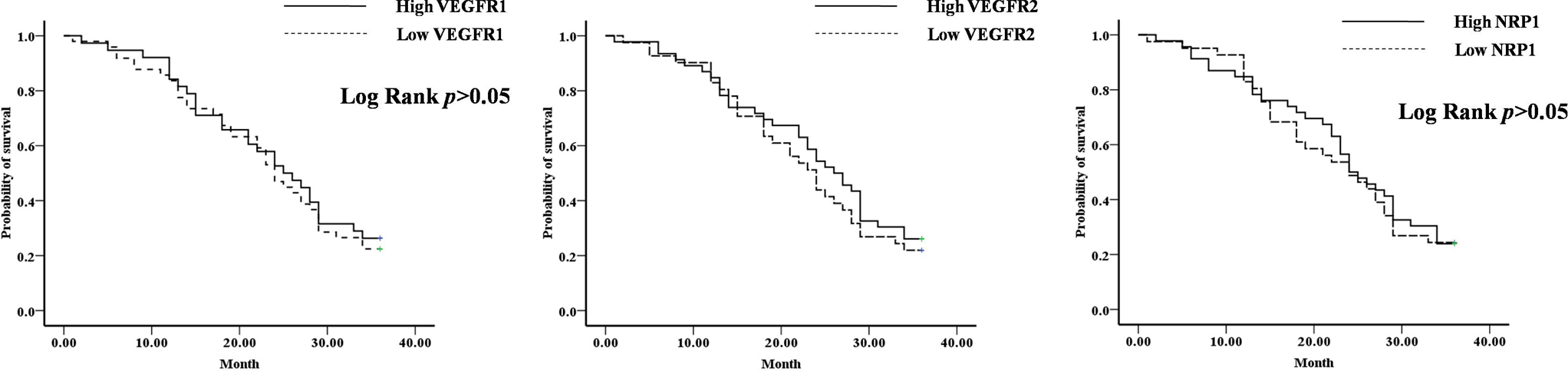

Supplement: Supplementary file 1 — supplement Fig1 [file 41419_2020_2428_MOESM1_ESM.tif]

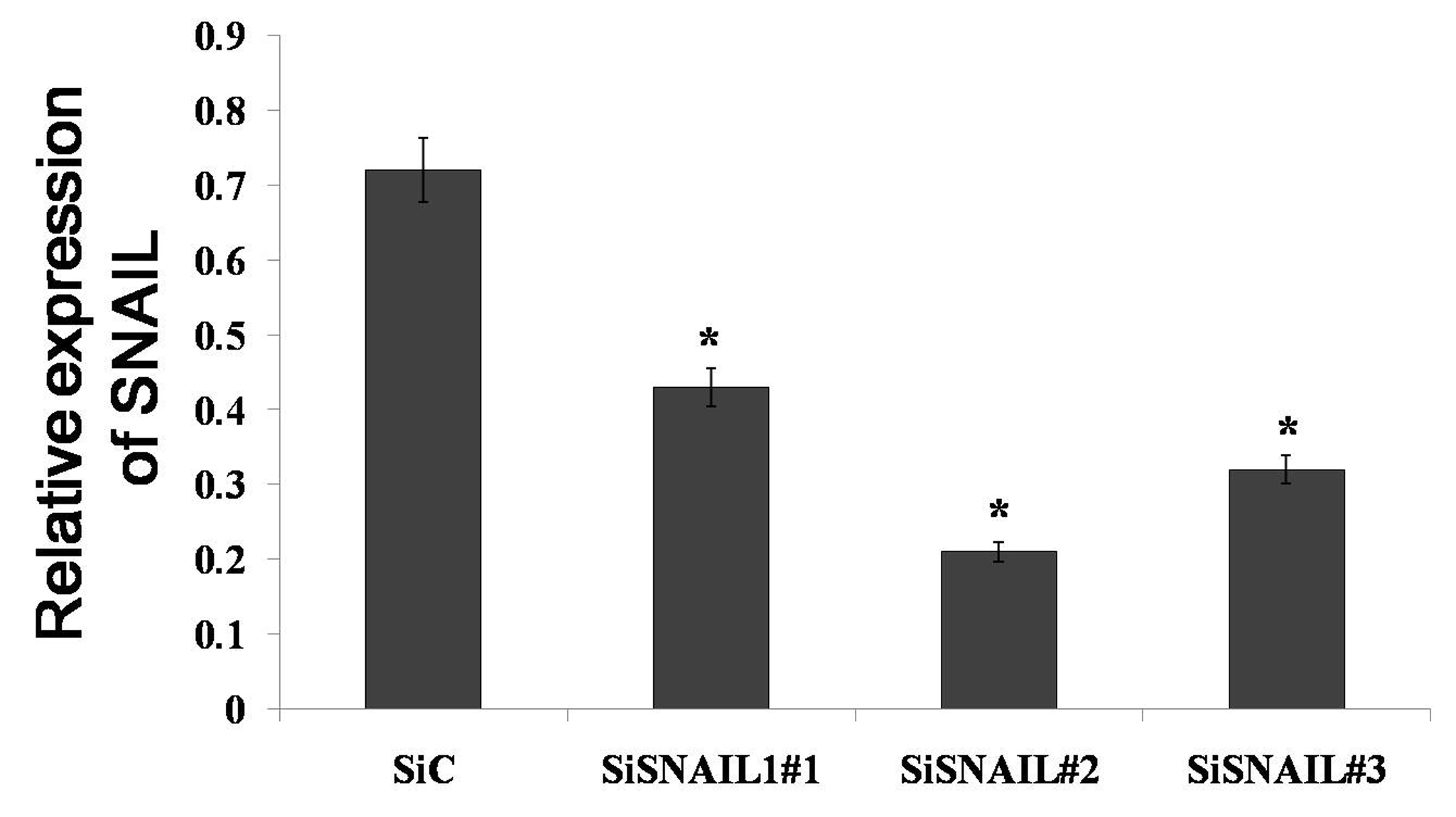

Supplement: Supplementary file 2 — supplement Fig2 [file 41419_2020_2428_MOESM2_ESM.tif]

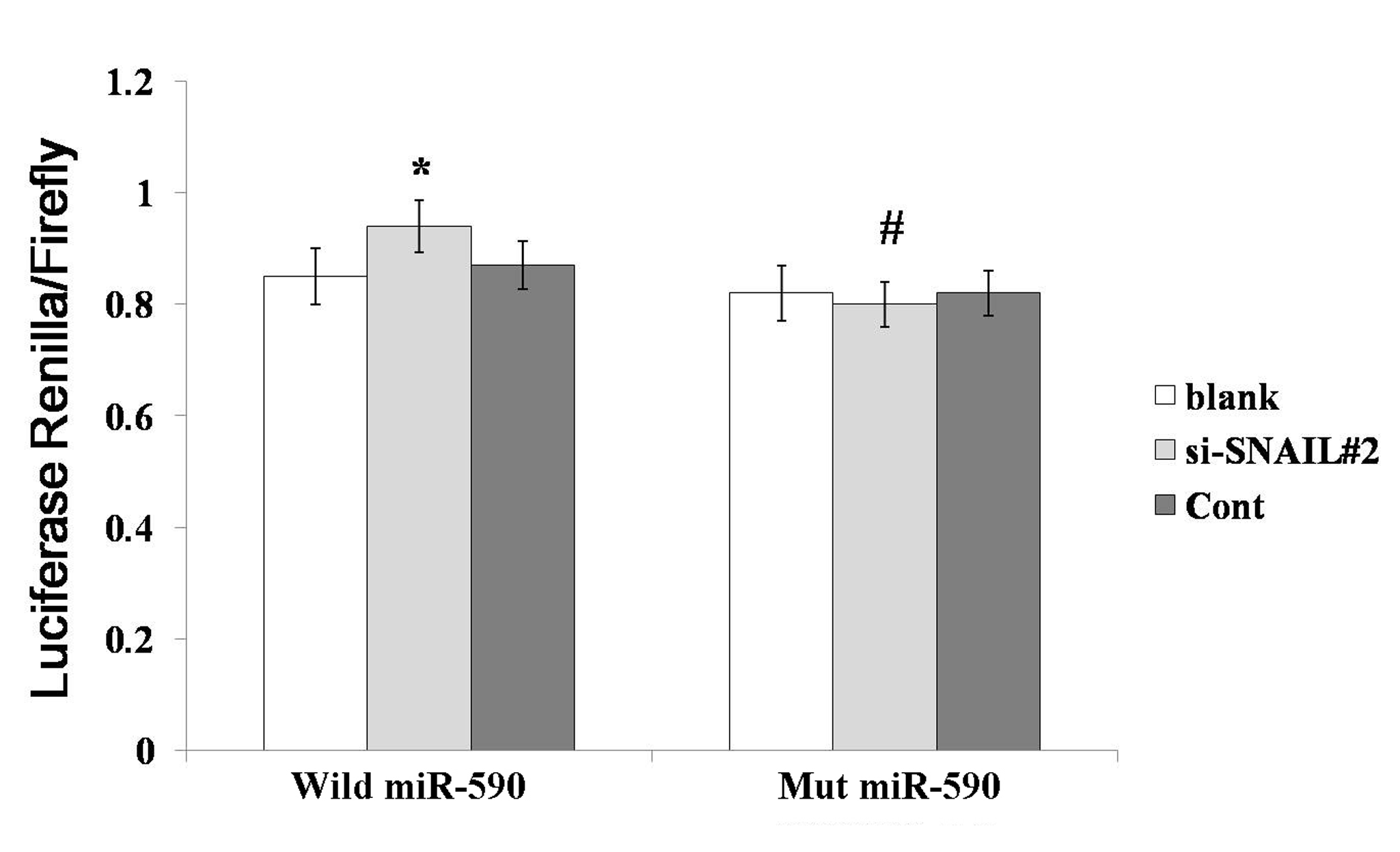

Supplement: Supplementary file 3 — supplement Fig3 [file 41419_2020_2428_MOESM3_ESM.tif]
