# Supplementary material for: The regulatory mechanism and biological significance of the Snail-miR590-VEGFR-NRP1 axis in the angiogenesis, growth and metastasis of gastric cancer
Source: Cell Death Dis. 2020 Apr 17;11(4):241. doi: 10.1038/s41419-020-2428-x (PMC7165172; doi:10.1038/s41419-020-2428-x)
Supplement: Supplementary file 5 — Supplementary legends [file 41419_2020_2428_MOESM5_ESM.docx]

**Supplemental Fig. 1 Kaplan-Meier analysis for overall survival based on VEGFR1/2 or NRP1 expression. The data are shown separately in human samples; * p<0.05.**

**Supplemental Fig. 2 SNAIL expression was detected by a western blot in AGS cells after treatment with 3 independent siRNA sequences (siNRP1) or a control (siC). SiSNAIL#2 was selected for the best knockout efficiency.**

**Supplemental Fig. 3 The si-SNAIL-expressing vector was cotransfected with a psiCHECK2 luciferase reporter plasmid, which contained a 3 kb fragment upstream of the human miR-590 stem-loop, into AGS cells. The luciferase activity was then observed.**

**Supplemental Tab.1 Predicted miRNAs that may regulate the expression of VEGFR1/2 or NRP1.**
